# Determinants of Self-Stigma in People with Parkinson’s Disease: A Mixed Methods Scoping Review

**DOI:** 10.3233/JPD-212869

**Published:** 2022-02-15

**Authors:** Anne-Marie Hanff, Anja K. Leist, Joëlle V. Fritz, Claire Pauly, Rejko Krüger, Margareta Halek, on behalf of the NCER-PD Consortium

**Affiliations:** a Transversal Translational Medicine, Luxembourg Institute of Health (LIH), Luxembourg; b Department of Social Sciences, University of Luxembourg, Esch-sur-Alzette, Luxembourg; c Parkinson Research Clinic (PRC), Centre Hospitalier de Luxembourg (CHL), Luxembourg; d Luxembourg Centre for Systems Biomedicine (LCSB), University of Luxembourg, Esch-sur-Alzette, Luxembourg; e Faculty of Health, School of Nursing, University Witten/Herdecke, Witten, Germany

**Keywords:** Parkinson’s disease, social stigma, shame, social discrimination, review, international classification of functioning, disability and health

## Abstract

**Background:**

Self-stigma in people with Parkinson’s disease (PD) can substantially impact quality of life and possibilities for social participation. An integrative analysis of determinants of self-stigma has been lacking.

**Objective:**

We sought to explore which complementary insights from qualitative and quantitative studies, as well as from expert consultation, could be gained.

**Methods:**

An established mixed methods study design was employed to first conduct a mixed methods scoping review of published qualitative and quantitative literature, and then consult with experts to arrive at an exhaustive list of determinants of self-stigma after a thematic synthesis.

**Results:**

A total of 87 unique determinants of self-stigma were identified. Quantitative studies and expert consultations mainly identified personal determinants of people with self-stigma (e.g., age, anxiety, or apathy). In contrast, qualitative studies identified social situations associated with self-stigma (e.g., joint meals of people with typical PD with others). Notably, self-stigma of people with PD was found to be particularly salient in unfamiliar places, at the working place or in contact with people without PD. Across methods, cognitive impairment, tremor, and abnormal walk and unsteady gait, respectively, were associated with self-stigma.

**Conclusion:**

The mixed method study design yielded complementary insights, but also factors commonly associated with self-stigma across methods. Future prioritization exercises may gain further insights into self-stigma of people with PD. Facilitating social encounters by both addressing needs of affected people and raising knowledge and public awareness may improve quality of life in people with PD.

## INTRODUCTION

The clinical symptoms of a person with typical Parkinson’s disease (PD) as described first by Dr. James Parkinson in 1817 include facial masking, the stooped posture, clenched and trembling arms, and shuffling gait [1]. These symptoms connote deformity and disability as the sociologist Nijhof [2] described in her research. Such typical PD manifestations are a root cause of stigmatization as they counteract social norms about normal behavior in a healthy social community [2]. More specifically, if in a social situation an unacquainted person shows behavior that deviates negatively from the expected stereotypical behavior of a healthy person, the behavior will be interpreted as sign of impairment. Typical PD symptoms can thus be considered as symbols associated with stigma if they are discrediting (discriminatory stimuli) and associated with a negative stereotype, for instance, if these symptoms correspond to typical behavior of a person under the influence of alcohol [3, 4]. The appearance (e.g., unsteady gate) is misattributed to be the result of alcohol misuse instead of a typical symptom of PD.

This process of stigmatization of PD symptoms can become internalized by the person affected by PD if they are aware of common misattributions of PD symptoms and exhibit or are anticipating exhibiting symptoms or behaviors commonly associated with undesirable stereotypes.

According to an international meta-analysis, self-stigma in people with typical PD was the dimension most significantly affected compared to the other dimensions of the Parkinson’s disease Questionnaire (PDQ39) [5]. Self-stigma has important consequences for overall quality of life and social participation for affected persons. In particular, self-stigma plays a significant role in determining whether and how effectively affected persons cope with typical PD [6]. Also, people with typical PD can feel incapable of meeting minimal social norms of interpersonal behavior which are important for creating and sustaining relationships with family, friends, coworkers, and others in their social environment [7]. Consequently, people with typical PD might withdraw from a public into a private world when they can no longer hide their symptoms [2, 8]. Notably, swallowing difficulties often induce isolation of people with typical PD as they may not be able to participate in joint meals which serve important social functions [9]. Self-stigma was reported as a determinant of depression [10] and as a key determinant of the quality of life of people with typical PD [8]. Moreover, a user-informed prioritization exercise confirmed self-stigma being one research priority defined by people with typical PD [11]. Thus, research on stigma has been identified as vital effort to enable people with typical PD to lead decent lives in the community [12]. Finally, knowledge on the underlying causes of stigma enables the development of effective nursing interventions to prevent or manage self-stigma of people with typical PD.

Despite numerous studies with qualitative and quantitative research designs, an integrative analysis of determinants of self-stigma in people with typical PD has been lacking. Therefore, the present study aims to summarize determinants of self-stigma, and sought to explore which complementary insights from qualitative and quantitative studies, as well as from expert consultation, could be gained regarding the determinants of self-stigma in people with PD [13]. Furthermore, the rationality of currently applied single-method approaches, i.e., literature review of quantitative studies will be explored. The following questions were addressed: What contextual determinants, that is, environmental and personal determinants and what body functions and -structure, activity and participation are associated with self-stigma in people with typical PD? Do qualitative and quantitative studies, and expert consultations, address different aspects of determinants of self-stigma?

## MATERIALS AND METHODS

According to the Classification of Functioning, Disability and Health (ICF)-framework, we defined *contextual determinants* as environmental determinants, that is, the physical, social and attitudinal environment in which people live and conduct their lives, *and personal determinants*, “which include gender, age, coping styles, social background, education, profession, past and current experience, overall behavior pattern, character and other determinants that influence how disability is experienced by the individual.” [14] We defined *body structure* as “anatomical parts of the body such organs, limbs and their components”, *activity* as the “execution of a task or action by an individual.”, and *participation* as “involvement in life situations.” [14]. The development of our mixed methods study design was based on previous work of the PARADISE (Psychosocial fActors Relevant to BrAin DISorders in Europe) consortium [15]. The PARADISE consortium applied a mixed methods study design to generate a pool of psychosocial difficulties and environmental determinants relevant for brain disorders.

### Mixed methods scoping review

According to the descriptive aim, the information about the risk of bias would not have influenced data synthesis. An assessment of methodological limitations or risk of bias of the evidence included within a scoping review is generally not performed [16]. Consequently, the method of a scoping review was chosen.

On 22 July 2020, we performed the mixed methods scoping review following the methodology of the JBI reviewer’s manual for mixed-methods systematic reviews [13] and scoping reviews [16] in the databases PubMed, CINAHL, and the Cochrane Library. No review protocol was registered in advance.

As recommended by Lizarondo et al. [13], a three-step search strategy was applied. An initial limited search for stigma and Parkinson’s disease in two databases (PubMed, CINAHL) identified no existing mixed methods literature reviews on the topic. This initial search was followed by an analysis of the words contained in the title and abstract of retrieved papers, and of the index terms of the articles to inform our search strategy [16]. Accordingly, MeSH and free-text terms (including spelling variants, synonyms, and truncation) were collected to identify appropriate search terms for the two search blocks typical PD and self-stigma. The following keywords for self-stigma were identified: Stigma^*^, Self Concept^*^, Self-Perception^*^, Perception^*^ self, Self Esteem^*^, embarrass^*^, shame, guilt^*^, humiliat^*^, degradat^*^, discredit^*^, disesteem, dishonor. The asterisk ^*^ replaces any number of characters. The truncation process captured variations, that is, terms with the same word stem (e.g., stigma, stigma*tization*, stigma*tisation*, stigma*tizing*, stigma*ta*, . . .). Search strategies were customized for each database. A search across all included databases using all keywords and index terms was done. Finally, we searched the reference lists of other reviews [17–20] for additional sources. The complete CINAHL search strategy is included in the Supplementary Material. The applied in- and exclusion criteria are illustrated in Table 1.

**Table 1:** In- and exclusion criteria in the mixed-methods scoping review

	Variable	Inclusion criteria	Exclusion criteria
Content	*p*	Population	Age of 18 years and more, Typical Parkinson’s disease diagnosed according to the MDS diagnostic criteria	Age of less than 18 years
				No diagnosis of typical PD
				No humans
	E	Exposure	Determinants associated with self-stigma Quantitative part: statistical significance (*p* < 0.05)	Articles not reporting the investigation of determinants associated with self-stigma
	O	Outcome	Self-stigma
Form	Design		Quantitative studies: Epidemiological studies measuring a statistical association between at least two variables (cohort, case-control-study, analytical cross-sectional study, RCTs, CCTs).	Articles without empirical data (editorials, reviews, protocols, and studies reporting the psychometric validation of an instrument).
			Qualitative studies: Studies that focus on qualitative data including, but not limited to, designs such as phenomenology, grounded theory, ethnography, and descriptive qualitative studies that describe the experience.
	Setting		No restrictions	No restrictions
	Time frame		No restrictions	No restrictions
	Language		No restrictions	No restrictions
	Culture		No restrictions	No restrictions

### Expert consultation

The authors suspected that patients were not aware of all determinants of self-stigma. Consequently, the clinical expert opinion of six clinicians, neuropsychologists, and PD nurses involved in the Luxembourg Parkinson Study and thus in daily contact with people with PD and their family and friends [21] was consulted according to the PARADISE consortium [15].

### Thematic synthesis

**Fig. 1:**
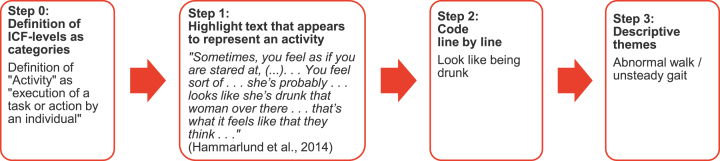
Exemplar process from “Definition of ICF-levels as categories” to “Assignment of ICF levels to descriptive themes” (thematic synthesis).

As illustrated in Fig. 1, we started thematic synthesis by predefining the categories. Operational definitions for each category were determined by the ICF-framework [14]. After reading the articles and highlighting all text that on first impression appears to represent a category, we coded all highlighted passages, i.e., defined line by line the keywords describing the meaning and content. In the third step, we looked for similarities and differences between the defined keywords. Finally, similar determinants were grouped and named by a descriptive theme.

### Data integration

**Fig. 2:**
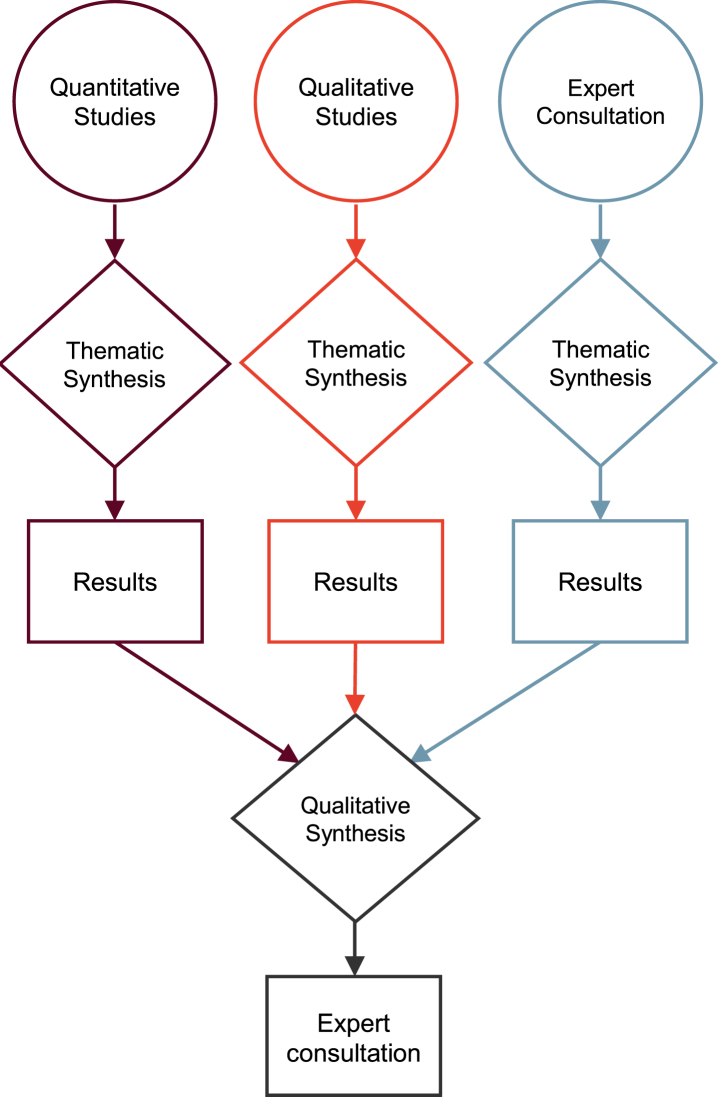
Results-based convergent mixed-methods synthesis design according to Hong et al. [25].

We performed a so-called *thematic synthesis* of the data [22] separately, before integrating the results of these syntheses in an overall *qualitative synthesis* in form of side-by-side comparisons. More specifically, we integrated the different sources according to a results-based convergent mixed-methods synthesis design [23] (Fig. 2). This approach allowed to determine whether the quantitative and qualitative data address different aspects of self-stigma and to determine in what ways the results confirm, disconfirm, or expand each other [24].

## RESULTS

**Fig. 3:**
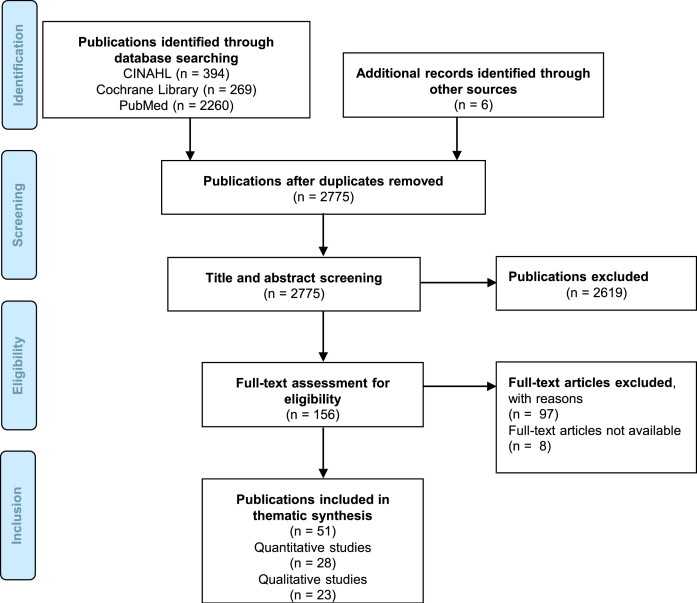
Study selection process for the mixed methods scoping review according to the PRISMA-ScR reporting guideline [25].

As Fig. 3 shows, after removing duplicates, we identified 28 quantitative [26–53] and 23 qualitative studies [2, 6, 8, 9, 54–68] through the mixed methods scoping review.

**Fig. 4:**
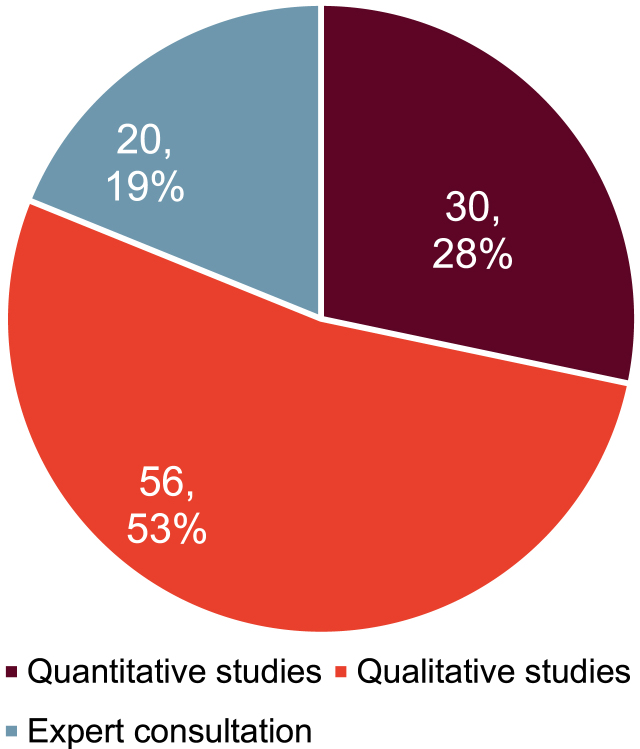
Number of determinants identified by data source.

As indicated in Fig. 4, in total 106 determinants were identified in the mixed methods study design. 19 determinants were identified by multiple data sources.

Quantitative cross-sectional studies were applying heterogenic definition and operationalization for determinants and the outcome. Only one quantitative study [46] investigated the self-stigma as a primary outcome.

**Fig. 5:**
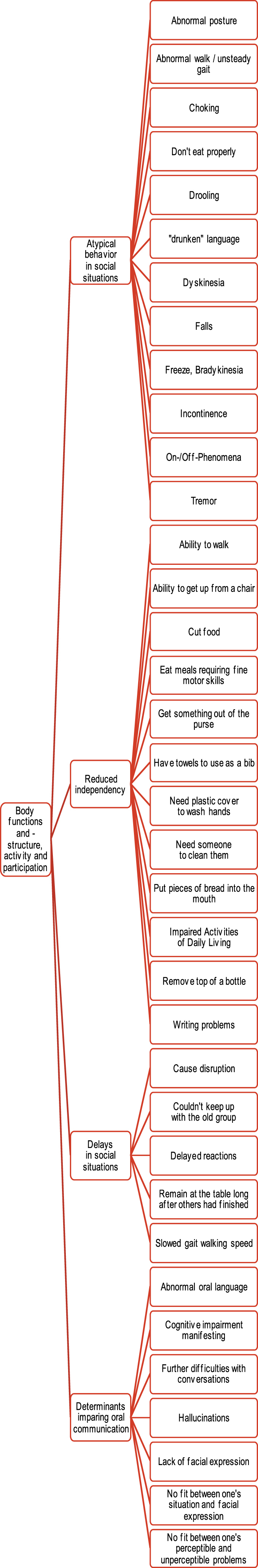
Body functions and structure, activity, and participation identified according to the ICF-framework [14].

**Fig. 6:**
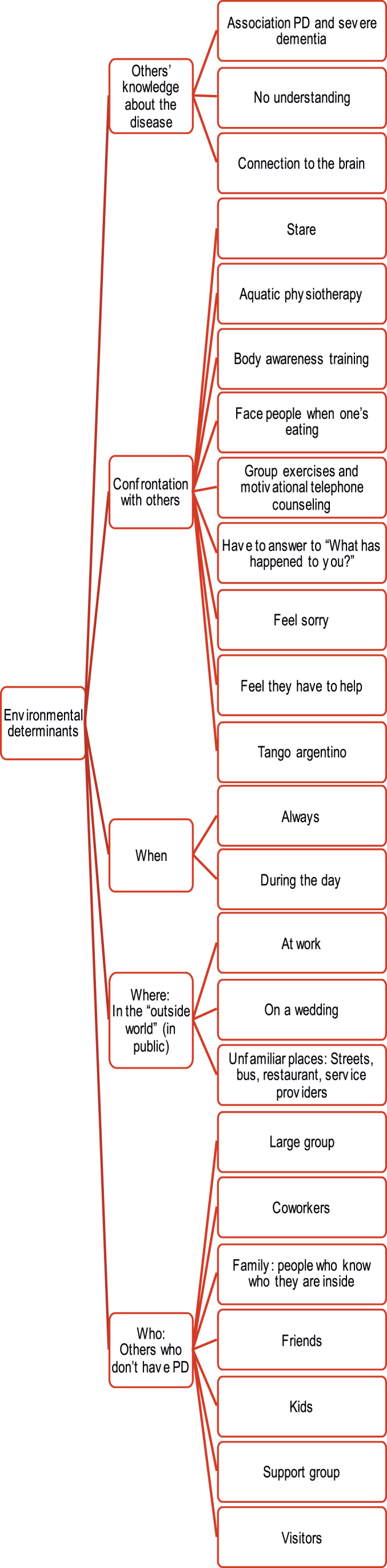
Environmental determinants identified according to the ICF-framework [14].

**Fig. 7:**
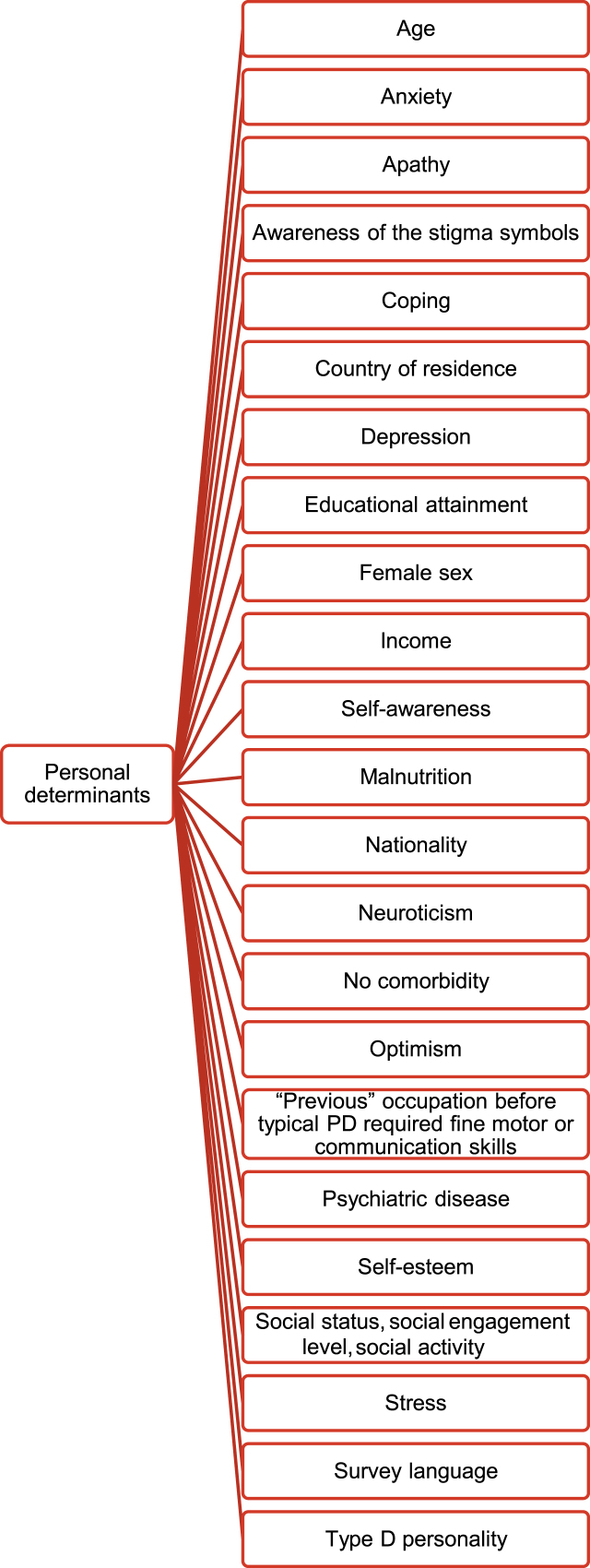
Personal determinants identified according to the ICF-framework [14].

Figures 5–7 synthesize identified determinants according to the ICF-framework [14].

To better understand the nature of self-stigma, common *misattributions of PD symptoms* were collected. Atypical behavior may be misinterpreted as:

• Intoxication from alcohol or street drugs [6, 58, 69];
• Expression of negative emotions, e.g., anger, nervousness, or, from the partner’s perspective, having fallen out of love [2, 58], grumpy, reticent [6, 66, 69];
• Expression of cognitive impairment or psychological disorder (e.g., depression) [9, 66, 68–70];
• Expression of a physical state, e.g., frailty, pain, being physically handicapped (e.g., from dyskinesia) [2, 8, 55, 58, 66, 69], there’s something wrong [55, 68];
• Socially deviant behavior (e.g., being a thief) [2, 66].

Tables 2–4 list all determinants of self-stigma and the source of data in a joint display.

**Table 2:** Identified body functions and structure, activity, and participation associated with self-stigma^1^

	Quantitative Studies									Qualitative Studies																						Expert consultation
	[43] (1x)	[30] (1x)	[40] (1x)	[49] (1x)	[47] (3x)	[29] (1x)	[37] (1x)	[45] (1x)	[28] (3x)	[55] (4x)	[56] (3x)	[57] (1x)	[59] (1x)	[61] (4x)	[63] (1x)	[65] (3x)	[67] (1x)	[71] (3x)	[72] (3x)	[60] (2x)	[6] (5x)	[58] (7x)	[9] (3x)	[62] (4x)	[64] (1x)	[2] (17x)	[66] (6x)	[68] (4x)	[8] (3x)	[20] (4x)	[70] (4x)	(5x)
**Atypical Behavior in Social Situations**
1. Abnormal posture (4x)^c,e^										X	X															X		X			
2. Abnormal walk/ unsteady gait (8x)^d,f^												X		X		X		X		X	X					X						X
3. Choking (1x)^c,e^																								X							
4. Don’t eat properly (1x)^c,e^																										X					
5. Drooling (3x)^d,f^	X																						X			X					
6. “drunken” language (1x)^b,e^																																X
7. Dyskinesia (6x)^d,f^							X		X	X						X										X			X		
8. Falls (2x)^c,e^														X							X										
9. Freeze, Bradykinesia (3x)^d,f^									X																		X	X			
10. Incontinence (2x)^b,c,d,e^															X																	X
11. On-/Off Phenomena (2x)^d,f^									X																		X				
12. Tremor (13x)^d,f^			X								X		X	X		X			X		X	X				X	X	X	X			X
**Reduced Independency**
13. Inability to walk (1x)^c,e^																										X					
14. Inability to get up from a chair (1x)^c,e^																										X					
15. Cut food (4x)^c,e^																			X		X					X	X				
16. Eat meals requiring fine motor skills (1x)^c,e^																										X					
17. Get something out of the purse (2x)^c,e^										X																	X				
18. Have towels to use as a bib (1x)^c,e^																										X					
19. Need plastic cover to wash hands (1x)^c,e^																													X		
20. Need someone to clean me (1x)^c,e^																		X													
21. Put pieces of bread into the mouth (1x)^c,e^																										X					
22. Impaired Activities of Daily Living (2x)^a^		X		X																											
23. Remove top of the bottle (1x)^c,e^										X																					
24. Writing problems (3x)^c,e^																						X				X	X				
**Delays in Social Situations**																															
25. Cause disruption (1x)^c,e^																								X							
26. Couldn’t keep up with the old group (3x)^c,e^														X					X												X
27. Delayed reactions (1x)^c,e^																						X									
28. Remain at the table long after others had finished (1x)^c,e^																							X								
29. Slowed gait (walking speed) (2x)^c,e^																						X				X					
**Determinants Impairing Oral Communication**																															
30. Abnormal oral language (5x)^d,f^					X																	X	X			X		X		X	
31. Cognitive impairment manifesting (7x)^d,f^					X	X		X										X						X	X					X		X
32. Further difficulties with conversations (3x)^d,f^					X																			X						X	
33. Hallucinations (2x)^c,e^																	X														X
34. Lack of facial expression (5x)^c,e^											X										X	X				X				X	
35. No fit between one’s situation and facial expression (3x)^c,e^																						X				X					X
36. No fit between own and other-perceived problems (2x)^c,e^																				X											X

1(... x) = Sum of identified studies / determinants per column / row, ^a^Quantitative studies only, ^b^Expert consultation only, ^c^Qualitative studies only, ^d^At least two different data and methods, ^e^Qualitative studies and / or expert consultation without confirmation by quantitative studies, ^f^Qualitative studies and / or expert consultation being confirmed by quantitative studies.

**Table 3:** Identified environmental determinants of self-stigma^1^

	Quantitative Studies							Qualitative Studies													Expert Consultation
Identified environmental determinants associated with self-stigma	[26] (1x)	[32] (1x)	[31] (1x)	[35] (1x)	[38] (1x)	[39] (1x)	[41] (1x)	[55] (2x)	[54] (1x)	[61] (1x)	[65] (1x)	[72] (5x)	[6] (3x)	[58] (4x)	[9] (1x)	[2] (6x)	[66] (8x)	[68] (5x)	[8] (2x)	[72] (1x)	(1x)
**Others’ Knowledge about the Disease**
37. Association between typical PD and severe dementia in the public’s eyes (1x)^c,e^																	X			
38. Others are not aware of symptoms of typical PD (3x)^c,e^											X	X		X						
39. People know it’s connected to the brain (1x)^c,e^																	X
**Social Situations**																				
40. All people stare at me (2x)^c,e^												X				X				
41. Aquatic physiotherapy (1x)^a^			X
42. Body awareness training (1x)^a^				X																
43. Face people when one’s eating (3x)^c,e^															X	X	X			
44. Group exercise and motivational telephone counseling (1x)^a^							X													
45. Have to answer “What has happened to you?” (1x)^c,e^																		X
46. People feel sorry for them (2)^c,e^												X					X			
47. People feel they have to help them (2x)^c,e^												X					X			
48. Tango Argentino (1x)^c,e^									X											
**When**
49. Always (1x)^c,e^														X						
50. Deep Brain Stimulation (1x)^a^		X																		
51. During the day (1x)^c,e^																X				
**Where: In the “Outside World”/ In Public**																				
52. At work (3x)^c,e^													X				X		X	
53. On a wedding (1x)^c,e^																		X		
54. Rural life settings (2x)^d,f^					X	X															X
55. Unfamiliar places, In the streets, Bus, Restaurant, Service providers (6x)^c,e^										X		X	X			X	X	X		
**Who: Others Who Don’t Have Typical PD**																				
56. A large group (1x)^c,e^																X				
57. Coworkers (4x)^c,e^													X				X	X	X	
58. Family: People who know who they are inside (1x)^c,e^								X												
59. Friends (1x)^c,e^																	X
60. Kids (2x)^c,e^								X						X
61. Neighbors (2x)^d,e^																				X	X
62. Support group (2x)^d,e^														X							X
63. Visitors (2x)^c,e^																X		X		
**Characteristics of the Rater of the PDQ-39**
64. Completion of the questionnaire by the caregiver (1x)^a^	X

^1^(... x)=Sum of identified studies / determinants per column / row, ^a^Quantitative studies only, ^b^Expert consultation only, ^c^Qualitative studies only, ^d^At least two different data and methods, ^e^Qualitative studies and / or expert consultation without confirmation by quantitative studies, ^f^Qualitative studies and / or expert consultation being confirmed by quantitative studies.

**Table 4:** Identified personal determinants of self-stigma^1^

	Quantitative Studies																		Expert Consultation
Identified personal determinants associated with self-stigma	[32] (1x)	[33] (1x)	[34] (1x)	[36] (1x)	[42] (1x)	[46] (2X)	[49] (7X)	[28] (2x)	[52] (10x)	[48] (1x)	[51] (2x)	[44] (1x)	[53] (2x)	[38] (1x)	[39] (1x)	[37] (1x)	[27] (1x)	[50] (1x)	(15x)
**Characteristics of the Person with Typical PD**
65. Age (3x)^a^						X		X	X									
66. Anxiety (2x)^d,f^							X												X
67. Apathy (1x)^b,e^																			X
68. Awareness of the stigma symbols (1x)^b,e^																			X
69. Coping (1x)^b,e^																			X
70. Country of residence (1x)^a^																X		
71. Depression (3x)^d,f^						X	X												X
72. Educational attainment (1x)^b,e^																			X
73. Female (2x)^d,f^									X										X
74. Income (1x)^b,e^																			X
75. Self-awareness (1x)^b,e^																			X
76. Malnutrition (2x)^a^				X	X													
77. Nationality (1x)^b,e^																			X
78. Neuroticism (1x)^a^			X															
79. No comorbidity (1x)^a^									X									
80. Optimism (1x)^a^							X											
81. (Previous) occupation before typical PD required fine motor skills or communication skills (1x)^b,e^																			X
82. Psychiatric disease (1x)^a^									X									
83. Self-esteem (1x)^a^							X											
84. Social status, social engagement level, social activity (1x)^b,e^																			X
85. Stress (1x)^a^							X											
86. Survey language (1x)^a^													X					
87. Type D personality (1x)^a^		X

1(... x) = Sum of identified studies / determinants per column / row, ^a^Quantitative studies only, ^b^Expert consultation only, ^c^Qualitative studies only, ^d^At least two different data and methods, ^e^Qualitative studies and / or expert consultation without confirmation by quantitative studies, ^f^Qualitative studies and / or expert consultation being confirmed by quantitative studies.

While quantitative studies identified most of the personal determinants, qualitative data revealed most of the subjective, patient-reported determinants related to the environment, body functions and structure, activity, and participation.

Most determinants were identified by qualitative studies only. Accordingly, 51%(44/87) of all determinants would not have been identified by excluding qualitative research. Furthermore, only 22%of the determinants (13/59) identified by qualitative studies and expert consultation were confirmed by quantitative studies.

Finally, three determinants were identified across all data and methods: Manifestation of cognitive impairment, tremor, and abnormal walk or unsteady gait, respectively.

## DISCUSSION

### Summary of findings

With a mixed methods scoping review of quantitative and qualitative studies, and expert consultation integrated with qualitative synthesis, a total of 87 determinants of self-stigma were identified.

As an overall finding, personal determinants (e.g., age, anxiety, or apathy) were identified by the quantitative studies and the expert consultation, while qualitative studies did not reveal any personal determinants. This supports our suspicion that patients are not always aware of personal determinants of self-stigma. In contrast, quantitative studies identified fewer symptoms and behaviors associated with self-stigma compared with qualitative findings.

As an insight from the qualitative studies, self-stigma arises from both other-perceived and only self-perceived symptoms and behaviors, thus, visible marks and invisible stains as described by Cumming and Cumming [74] are both important. This result confirms their theory stating self-stigma acquires its meaning through the emotion it generates within the person bearing it and the feeling and behavior toward him. Nijhof [2] concluded that self-stigma is always perceived in contact with the “outside world”, that is, with people who are not part of the family. Similarly, our work identified social situations associated with self-stigma, for example, joint meals of people with typical PD with others, and the person with PD perceiving the other person as feeling sorry for them, staring at them (questioningly). In particular, self-stigma is salient in unfamiliar places and at the working place. Furthermore, people with typical PD are perceiving self-stigma when they are around persons who don’t have typical PD like friends, visitors, neighbors, coworkers, service providers, or children. Consequently, our results confirm statements by Major and O’Brien [73] and Cumming and Cumming [74]: Self-stigma originates in the social context instead in the person, i.e., is reinforced in interaction with others.

### Implications for personalized interventions and awareness campaigns

As the PD-related knowledge of others in social encounters or in public is an important factor associated with self-stigma, the present results can be integrated in communication campaigns informing about the identified factors (e.g., impaired communication) to increase awareness about typical symptoms in PD. Further, personalized support for people with PD may also be helpful to reduce self-stigma. Support groups like Parkinson Associations seem to be an important resource for people with typical PD and should be facilitated. Communication guidance enabling people with PD to speak more openly about their symptoms with others may facilitate social encounters with unacquainted persons and being in the “outside world”.

### Strengths and limitations of the study

Previous reviews of qualitative studies [17–20] described experiences of self-stigma by people with typical PD. The integration of qualitative and quantitative research is one strength of the present study. Factors that were identified through more than one method (qualitative study, quantitative study, or expert consultation) can be considered to be validated through this approach. Other factors that were identified by only one of the methods may merit attention also through other research methods. More than 50%of all factors would not have been identified by excluding qualitative research, showing the importance of the consideration of multiple sources and types of data when creating an overview of the determinants of self-stigma. The need for further quantitative investigations of determinants of self-stigma is highlighted by the fact that less than 20%of all factors identified by qualitative studies and expert consultation were confirmed by quantitative studies. Furthermore, the detailed documentation of our review enables reproducibility, whereas some of the recent reviews, such as Angulo et al. [18] have preferred to review the literature in a narra-tive way.

The mixed methods study design employed here is less exhaustive than other forms of research [16], however it has enabled us to arrive at an important map of existing evidence on determinants of self-stigma. Thus, the applied criteria of a mixed methods scoping review described by Booth and colleagues [75] and [16] are less rigorous than those of classical systematic reviews (i.e., no formal quality assessment, no grey literature, no search for unpublished studies). The restricted use of published data for the thematic synthesis of qualitative studies might have resulted in a lack of identification of some factors. Adding the method of expert consultation however further enriched the collection of evidence through identification of further unique factors.

From the qualitative synthesis, it remains unclear which of the several identified factors are most strongly related to self-stigma, as a prioritization of factors should be informed by persons living with PD and their caregivers. Consequently, further systematic prioritization of the identified determinants of the self-stigma should be investigated in future studies. In addition, typical misattributions related to the three motor- and non-motor symptoms (i.e., manifestation of cognitive impairment, tremor, and abnormal walk/unsteady gait) that were identified across all methods need to be explored by further research.

From the qualitative synthesis, factors that were only identified by qualitative research or expert consultation would merit further attention in quantitative research in order to better understand the phenomenon of self-stigma. For example, personal characteristics such as self-awareness, and demographic characteristics such as income and thus possible health inequities in self-stigma, could inform interventions. The present study, through the comprehensive collection of determinants of self-stigma, provided an opportunity to gain a better understanding of the phenomenon of self-stigma. The mixed method study design combined with expert consultation yielded complementary insights, but also common factors that were associated with self-stigma across methods. Future research may gain further insights into the differential importance of these factors by mixed-method and user-informed studies to allow a prioritization of the numerous identified determinants of self-stigma. Facilitating social encounters by both addressing needs of affected people and raising knowledge and awareness of the public may reduce self-stigma and improve quality of life in people with PD.

## Supplementary Material

Supplementary Material

## References

[1] Parkinson J (2002) An essay on the shaking palsy. 1817. J Neuropsychiatry Clin Neurosci 14, 223–236; discussion 222. 11983801 10.1176/jnp.14.2.223

[2] Nijhof G (1995) Parkinson’s disease as a problem of shame in public appearance. Sociol Health Illn 17, 193–205.

[3] Goffman E (1991) Stigma and social identity. In Stigma. Notes on the management of spoiled identity, Goffman E, ed. Simon & Schuster, New York.

[4] Link BG , Phelan JC (2001) Conceptualizing stigma. Annu Rev School 27, 363–385.

[5] Soh SE , McGinley JL , Watts JJ , Iansek R , Morris ME (2012) Health-related quality of life of Australians with Parkinson disease: a comparison with international studies. Physiother Can 64, 338–346. 23997388 10.3138/ptc.2011-26 PMC3484904

[6] Chiong-Rivero H , Ryan GW , Flippen C , Bordelon Y , Szumski NR , Zesiewicz TA , Vassar S , Weidmer B , Garcia RE , Bradley M , Vickrey BG (2011) Patients’ and caregivers’ experiences of the impact of Parkinson’s disease on health status. Patient Relat Outcome Meas 2011, 57–70. 21691459 10.2147/PROM.S15986 PMC3117663

[7] Tickle-Degnen L , Saint-Hilaire M , Thomas CA , Habermann B , Martinez LS , Terrin N , Noubary F , Naumova EN (2014) Emergence and evolution of social self-management of Parkinson’s disease: study protocol for a 3-year prospective cohort study. BMC Neurol 14, 95. 24885181 10.1186/1471-2377-14-95 PMC4016672

[8] Soleimani MA , Bastani F , Negarandeh R , Greysen R (2016) Perceptions of people living with Parkinson’s disease: a qualitative study in Iran. Br J Community Nurs 21, 188–195. 27282505 10.12968/bjcn.2016.21.4.188

[9] Miller N , Noble E , Jones D , Burn D (2006) Hard to swallow: dysphagia in Parkinson’s disease. Age Ageing 35, 614–618. 17047007 10.1093/ageing/afl105

[10] Schrag A , Jahanshami M , Quinn NP (2001) What contributes to depression in Parkinson’s disease? Psychol Med 31, 65–73. 11200961 10.1017/s0033291799003141

[11] Schipper K , Dauwerse L , Hendrikx A , Leedekerken JW , Abma TA (2014) Living with Parkinson’s disease: priorities for research suggested by patients. Parkinsonism Relat Disord 20, 862–866. 24874526 10.1016/j.parkreldis.2014.04.025

[12] Pescosolido BA , Martin JK , Lang A , Olafsdottir S (2008) Rethinking theoretical approaches to stigma: a Framework Integrating Normative Influences on Stigma (FINIS). Soc Sci Med 67, 431–440. 18436358 10.1016/j.socscimed.2008.03.018 PMC2587424

[13] Lizarondo L , Stern C , Carrier J , Godfrey C , Rieger K , Salmond S , Apostolo J , Kirkpatrick P , Loveday H (2019) Chapter 8: Mixed methods systematic reviews. In Joanna Brigs Institute Reviewer’s Manual, Aromataris E, Munn Z, eds. The Joanna Brigs Institute.

[14] World Health Organization (2002) Towards a Common Language for Functioning, Disability and Health. ICF The International Classification of Functioning, Disability and Health. World Health Organization, Geneva.

[15] Cieza A , Anczewska M , Ayuso-Mateos JL , Baker M , Bickenbach J , Chatterji S , Hartley S , Leonardi M , Pitkanen T , Consortium P (2015) Understanding the impact of brain disorders: towards a ‘horizontal epidemiology’ of psychosocial difficulties and their determinants. PLoS One 10, e0136271. 26352911 10.1371/journal.pone.0136271 PMC4564202

[16] Peters M , Godfrey C , McInerney P , Munn Z , Tricco A , Khalil H (2020) Chapter 11: Scoping reviews. In Joanna Briggs Institute Reviewers’ Manual, Aromataris E, Munn Z, eds. Joanna Briggs Institute.

[17] Maffoni M , Giardini A , Pierobon A , Ferrazzoli D , Frazzitta G (2017) Stigma experienced by Parkinson’s disease patients: a descriptive review of qualitative studies. Parkinsons Dis 2017, 7203259. 28243481 10.1155/2017/7203259 PMC5294385

[18] Angulo J , Fleury V , Péron JA , Penzenstadler L , Zullino D , Krack P (2019) Shame in Parkinson’s disease: a review. J Parkinsons Dis 9, 489–499. 31081792 10.3233/JPD-181462 PMC6700625

[19] Mai T , Schnepp W , Hohmann U (2010) The situation of people suffering from Parkinson’s disease and their relatives in the mirror of literature - an overview. Pflege 23, 81–98. 20361406 10.1024/1012-5302/a000021

[20] Soundy A , Stubbs B , Roskell C (2014) The experience of Parkinson’s disease: a systematic review and meta-ethnography. ScientificWorldJournal 2014, 613592. 25525623 10.1155/2014/613592 PMC4265687

[21] Hipp G , Vaillant M , Diederich NJ , Roomp K , Satagopam VP , Banda P , Sandt E , Mommaerts K , Schmitz SK , Longhino L , Schweicher A , Hanff AM , Nicolai B , Kolber P , Reiter D , Pavelka L , Binck S , Pauly C , Geffers L , Betsou F , Gantenbein M , Klucken J , Gasser T , Hu MT , Balling R , Kruger R (2018) The Luxembourg Parkinson’s Study: a comprehensive approach for stratification and early diagnosis. Front Aging Neurosci 10, 326. 30420802 10.3389/fnagi.2018.00326 PMC6216083

[22] Thomas J , Harden A (2008) Methods for the thematic synthesis of qualitative research in systematic reviews. BMC Med Res Methodol 8, 45. 18616818 10.1186/1471-2288-8-45 PMC2478656

[23] Hong QN , Pluye P , Bujold M , Wassef M (2017) Convergent and sequential synthesis designs: implications for conducting and reporting systematic reviews of qualitative and quantitative evidence. Syst Rev 6, 61. 28335799 10.1186/s13643-017-0454-2 PMC5364694

[24] Creswell JW , Plano Clark VL (2018) Designing and conducting mixed methods research, Sage, Los Angeles.

[25] Tricco AC , Lillie E , Zarin W , O’Brien KK , Colquhoun H , Levac D , Moher D , Peters MDJ , Horsley T , Weeks L , Hempel S , Akl EA , Chang C , McGowan J , Stewart L , Hartling L , Aldcroft A , Wilson MG , Garritty C , Lewin S , Godfrey CM , Macdonald MT , Langlois EV , Soares-Weiser K , Moriarty J , Clifford T , Tuncalp O , Straus SE (2018) PRISMA Extension for Scoping Reviews (PRISMA-ScR): checklist and explanation. Ann Intern Med 169, 467–473. 30178033 10.7326/M18-0850

[26] Balash Y , Korczyn AD , Knaani J , Migirov AA , Gurevich T (2017) Quality-of-life perception by Parkinson’s disease patients and caregivers. Acta Neurol Scand 136, 151–154. 28083960 10.1111/ane.12726

[27] Cano-de-la-Cuerda R , Vela-Desojo L , Miangolarra-Page JC , Macías-Macías Y , Muñoz-Hellín E (2011) Axial rigidity and quality of life in patients with Parkinson’s disease: a preliminary study. Qual Life Res 20, 817–823. 21170683 10.1007/s11136-010-9818-y

[28] Chapuis S , Ouchchane L , Metz O , Gerbaud L , Durif F (2005) Impact of the motor complications of Parkinson’s disease on the quality of life. Mov Disord 20, 224–230. 15384126 10.1002/mds.20279

[29] Corallo F , De Cola MC , Lo Buono V , Di Lorenzo G , Bramanti P , Marino S (2017) Observational study of quality of life of Parkinson’s patients and their caregivers. Psychogeriatrics 17, 97–102. 27338524 10.1111/psyg.12196

[30] da Silva AG , Leal VP , da Silva PR , Freitas FC , Linhares MN , Walz R , Malloy-Diniz LF , Diaz AP , Palha AP (2020) Difficulties in activities of daily living are associated with stigma in patients with Parkinson’s disease who are candidates for deep brain stimulation. Braz J Psychiatry 42, 190–194. 31389495 10.1590/1516-4446-2018-0333 PMC7115448

[31] da Silva DM , Nunes MCO , Oliveira PJdAL , Coriolano MdGWdS , Berenguer FdA , Lins OG , Ximenes DKG (2013) Effects of aquatic physiotherapy on life quality on subjects with Parkinson disease. Fisioterapia Pesquisa 20, 17–23.

[32] Dafsari HS , Reker P , Stalinski L , Silverdale M , Rizos A , Ashkan K , Barbe MT , Fink GR , Evans J , Steffen J , Samuel M , Dembek TA , Visser-Vandewalle V , Antonini A , Ray-Chaudhuri K , Martinez-Martin P , Timmermann L , EUROPAR and the IPMDS (International Parkinson’s and Movement Disorders Society) Non-Motor Parkinson’s Disease Study Group (2018) Quality of life outcome after subthalamic stimulation in Parkinson’s disease depends on age. Mov Disord 33, 99–107. 29150860 10.1002/mds.27222

[33] Dubayova T , Nagyova I , Havlikova E , Rosenberger J , Gdovinova Z , Middel B , van Dijk JP , Groothoff JW (2009) The association of type D personality with quality of life in patients with Parkinson’s disease. Aging Mental Health 13, 905–912. 19888711 10.1080/13607860903046529

[34] Dubayova T , Nagyova I , Havlikova E , Rosenberger J , Gdovinova Z , Middel B , van Dijk JP , Groothoff JW (2009) Neuroticism and extraversion in association with quality of life in patients with Parkinson’s disease. Qual Life Res 18, 33–42. 18989757 10.1007/s11136-008-9410-x

[35] Ghielen I , van Wegen EEH , Rutten S , de Goede CJT , Houniet-de Gier M , Collette EH , Burgers-Bots IAL , Twisk JWR , Kwakkel G , Vermunt K , van Vliet B , Berendse HW , van den Heuvel OA (2017) Body awareness training in the treatment of wearing-off related anxiety in patients with Parkinson’s disease: Results from a pilot randomized controlled trial. J Psychosom Res 103, 1–8. 29167034 10.1016/j.jpsychores.2017.09.008

[36] Gruber MT , Witte OW , Grosskreutz J , Prell T (2020) Association between malnutrition, clinical parameters and health-related quality of life in elderly hospitalized patients with Parkinson’s disease: A cross-sectional study. PLoS One 15, e0232764. 32365092 10.1371/journal.pone.0232764 PMC7197805

[37] Hechtner MC , Vogt T , Zollner Y , Schroder S , Sauer JB , Binder H , Singer S , Mikolajczyk R (2014) Quality of life in Parkinson’s disease patients with motor fluctuations and dyskinesias in five European countries. Parkinsonism Relat Disord 20, 969–974. 24953743 10.1016/j.parkreldis.2014.06.001

[38] Hristova DR , Hristov JI , Mateva NG , Papathanasiou JV (2009) Quality of life in patients with Parkinson’s disease. Folia Med (Plovdiv) 51, 58–64. 20232661

[39] Klepac N , Pikija S , Kraljic T , Relja M , Trkulja V , Juren S , Pavlicek I , Babic T (2007) Association of rural life setting and poorer quality of life in Parkinson’s disease patients: a cross-sectional study in Croatia. Eur J Neurol 14, 194–198. 17250729 10.1111/j.1468-1331.2006.01604.x

[40] Lageman SK , Cash TV , Mickens MN (2014) Patient-reported needs, non-motor symptoms, and quality of life in essential tremor and Parkinson’s disease. Tremor Other Hyperkinet Mov (N Y) 4, 240. 24932425 10.7916/D8RF5S4J PMC4050172

[41] Lee J , Choi M , Yoo Y , Ahn S , Jeon JY , Kim JY , Byun JY (2017) Impacts of an exercise program and motivational telephone counseling on health-related quality of life in people with Parkinson’s disease. Rehabilitation Nursing 44, 161–170. 10.1097/rnj.0000000000000106 29345633

[42] Ongun N (2018) Does nutritional status affect Parkinson’s Disease features and quality of life? PLoS One 13, e0205100. 30278074 10.1371/journal.pone.0205100 PMC6168151

[43] Ou R , Guo X , Wei Q , Cao B , Yang J , Song W , Shao N , Zhao B , Chen X , Shang H (2015) Prevalence and clinical correlates of drooling in Parkinson disease: a study on 518 Chinese patients. Parkinsonism Relat Disord 21, 211–215. 25537930 10.1016/j.parkreldis.2014.12.004

[44] Peto V , Fitzpatrick R , Jenkinson C (1997) Self-reported health status and access to health services in a community sample with Parkinson’s disease. Disabil Rehabil 19, 97–103. 9134352 10.3109/09638289709166833

[45] Reginold W , Duff-Canning S , Meaney C , Armstrong MJ , Fox S , Rothberg B , Zadikoff C , Kennedy N , Gill D , Eslinger P , Marshall F , Mapstone M , Chou KL , Persad C , Litvan I , Mast B , Tang-Wai D , Lang AE , Marras C (2013) Impact of mild cognitive impairment on health-related quality of life in Parkinson’s disease. Dement Geriatr Cogn Disord 36, 67–75. 23774742 10.1159/000350032

[46] Salazar RD , Weizenbaum E , Ellis TD , Earhart GM , Ford MP , Dibble LE , Cronin-Golomb A (2019) Predictors of self-perceived stigma in Parkinson’s disease. Parkinsonism Relat Disord 60, 76–80. 30297211 10.1016/j.parkreldis.2018.09.028 PMC6433539

[47] Schalling E , Johansson K , Hartelius L (2018) Speech and communication changes reported by people with Parkinson’s disease. Folia Phoniatr Logop 69, 131–141. 10.1159/000479927 29346787

[48] Schrag A , Hovris A , Morley D , Quinn N , Jahanshahi M (2003) Young- versus older-onset Parkinson’s disease: impact of disease and psychosocial consequences. Mov Disord 18, 1250–1256. 14639664 10.1002/mds.10527

[49] Simpson J , Lekwuwa G , Crawford T (2014) Predictors of quality of life in people with Parkinson’s disease: evidence for both domain specific and general relationships. Disabil Rehabil 36, 1964–1970. 24499208 10.3109/09638288.2014.883442

[50] Song W , Guo X , Chen K , Chen X , Cao B , Wei Q , Huang R , Zhao B , Wu Y , Shang HF (2014) The impact of non-motor symptoms on the Health-Related Quality of Life of Parkinson’s disease patients from Southwest China. Parkinsonism Relat Disord 20, 149–152. 24161377 10.1016/j.parkreldis.2013.10.005

[51] Tomic S , Rajkovaca I , Pekic V , Salha T , Misevic S (2017) Impact of autonomic dysfunctions on the quality of life in Parkinson’s disease patients. Acta Neurol Belg 117, 207–211. 28028676 10.1007/s13760-016-0739-6

[52] Wu Y , Guo XY , Wei QQ , Song W , Chen K , Cao B , Ou RW , Zhao B , Shang HF (2014) Determinants of the quality of life in Parkinson’s disease: results of a cohort study from Southwest China. J Neurol Sci 340, 144–149. 24679837 10.1016/j.jns.2014.03.014

[53] Zhao YJ , Tan LC , Lau PN , Au WL , Li SC , Luo N (2008) Factors affecting health-related quality of life amongst Asian patients with Parkinson’s disease. Eur J Neurol 15, 737–742. 18494793 10.1111/j.1468-1331.2008.02178.x

[54] Beerenbrock Y , Meyer L , Böhme J , Herrlich S , Mews S , Berger B , Martin D , Büssing A (2020) Perceived effects of Tango Argentino on body experience in persons with Parkinson’s disease (PD)-A qualitative study with affected persons and their partners. Complement Ther Med 48, 102221. 31987239 10.1016/j.ctim.2019.102221

[55] Bramley N , Eatough V (2005) The experience of living with Parkinson’s disease: An interpretative phenomenological analysis case study. Psychol Health 20, 223–235.

[56] Fleming V , Tolson D , Schartau E (2004) Changing perceptions of womanhood: living with Parkinson’s disease. Int J Nurs Stud 41, 515–524. 15120980 10.1016/j.ijnurstu.2003.12.004

[57] Hammarlund CS , Andersson K , Andersson M , Nilsson MH , Hagell P (2014) The significance of walking from the perspective of people with Parkinson’s disease. J Parkinsons Dis 4, 657–663. 25147140 10.3233/JPD-140399

[58] Hermanns M (2013) The invisible and visible stigmatization of Parkinson’s disease. J Am Assoc Nurse Pract 25, 563–566. 24170489 10.1111/1745-7599.12008

[59] Heusinkveld LE , Hacker ML , Turchan M , Davis TL , Charles D (2018) Impact of tremor on patients with early stage Parkinson’s disease. Front Neurol 9, 628. 30123178 10.3389/fneur.2018.00628 PMC6085452

[60] Maffoni M , Pierobon A , Frazzitta G , Callegari S , Giardini A (2019) Living with Parkinson’s—past, present and future: a qualitative study of the subjective perspective. Br J Nurs 28, 764–771. 31242113 10.12968/bjon.2019.28.12.764

[61] Marr JA (1991) The experience of living with Parkinson’s disease. J Neurosci Nurs 23, 325–329. 1835998 10.1097/01376517-199110000-00010

[62] Miller N , Noble E , Jones D , Burn D (2006) Life with communication changes in Parkinson’s disease. Age Ageing 35, 235–239. 16540492 10.1093/ageing/afj053

[63] Moriarty HJ , Robinson JP , Bunting-Perry L , Bradway CW (2016) Cognitive, affective, and behavioral dimensions of the lower urinary tract symptom experience in men with Parkinson’s disease. J Wound Ostomy Continence Nurs 43, 80–87. 26727686 10.1097/WON.0000000000000165

[64] Mshana G , Dotchin CL , Walker RW (2011) ‘We call it the shaking illness’: perceptions and experiences of Parkinson’s disease in rural northern Tanzania. BMC Public Health 11, 219–219. 21477284 10.1186/1471-2458-11-219 PMC3088907

[65] Nazzal MS , Khalil H (2017) Living with Parkinson’s disease: A Jordanian perspective. Scand J Occup Ther 24, 74–82. 27677343 10.1080/11038128.2016.1234643

[66] Posen J , Moore O , Tassa DS , Ginzburg K , Drory M , Giladi N (2000) Young women with PD: a group work experience. Soc Work Health Care 32, 77–91. 11291893 10.1300/J010v32n01_06

[67] Renouf S , ffytche D , Pinto R , Murray J , Lawrence V (2018) Visual hallucinations in dementia and Parkinson’s disease: A qualitative exploration of patient and caregiver experiences. Int J Geriatr Psychiatry 33, 1327–1334. 29953689 10.1002/gps.4929

[68] Soleimani MA , Negarandeh R , Bastani F , Greysen R (2014) Disrupted social connectedness in people with Parkinson’s disease. Br J Community Nurs 19, 136–141. 24897835 10.12968/bjcn.2014.19.3.136

[69] Mersch-Mahowald S , (o.J.) Unser Leben mit Parkinson... und wie es vorher war. Erfahrungen, Gefühle und Wünsche von 52 Betroffenen., Luxembourg.

[70] Sunvisson H , Ekman S-L (2001) Environmental influences on the experiences of people with Parkinson’s disease. Nurs Inq 8, 41–50. 11882200 10.1046/j.1440-1800.2001.00089.x

[71] Vann-Ward T , Morse JM , Charmaz K (2017) Preserving self: theorizing the social and psychological processes of living with Parkinson disease. Qual Health Res 27, 964–982. 28818020 10.1177/1049732317707494

[72] Valcarenghi RV , Alvarez AM , Siewert JS , Tomasi AVR , Costa Santos SS , Lopes Nunes SF (2018) The daily lives of people with Parkinson’s disease. Rev Bras Enfermagem 71, 272–279. 10.1590/0034-7167-2016-0577 29412283

[73] Major B , O’Brien LT (2005) The social psychology of stigma. Annu Rev Psychol 56, 393–421. 15709941 10.1146/annurev.psych.56.091103.070137

[74] Cumming J , Cumming E (1965) On the stigma of mental illness. Community Ment Health J 1, 135–143.

[75] Grant MJ , Booth A (2009) A typology of reviews: an analysis of 14 review types and associated methodologies. Health Info Libr J 26, 91–108. 19490148 10.1111/j.1471-1842.2009.00848.x

