# Supplementary material for: Determinants of Self-Stigma in People with Parkinson’s Disease: A Mixed Methods Scoping Review
Source: J Parkinsons Dis. 2022 Feb 15;12(2):509–22. doi: 10.3233/JPD-212869 (PMC8925108; doi:10.3233/JPD-212869)
Supplement: Supplementary Material [file jpd-12-jpd212869-s001.pdf]

Supplementary Material

Determinants of Self-Stigma in People with Parkinson's Disease: A Mixed Methods Scoping Review

CINAHL Search Strategy

ID	Command	Results 22.07.2020 13:53
1.	(MH "Parkinson Disease") OR "parkinson's disease"	26687
2.	(MH "Stigma") OR "stigma"	22354
3.	shame OR discrimination OR prejudice OR stereotyp* OR dishonor OR disesteem OR discredit* OR degradat* OR humiliat* OR guilt* OR embarrass*	71594
4.	(MH "Stereotyping")	6541
5.	(MH "Ageism") OR (MH "Prejudice")	7636
6.	(MH "Discrimination")	11139
7.	(MH "Shame")	2113
8.	S2 OR S3 OR S4 OR S5 OR S6 OR S7	89856
**9.**	**S1 AND S8**	**394**
